# Dietary agent, benzyl isothiocyanate inhibits signal transducer and activator of transcription 3 phosphorylation and collaborates with sulforaphane in the growth suppression of PANC-1 cancer cells

**DOI:** 10.1186/1475-2867-9-24

**Published:** 2009-08-27

**Authors:** Brian Hutzen, William Willis, Sarah Jones, Ling Cen, Stephanie Deangelis, Beng Fuh, Jiayuh Lin

**Affiliations:** 1 Department of Pediatrics, The Research Institute at Nationwide Childrens' Hospital, Columbus, OH, USA; 2 Molecular, Cellular, and Developmental Biology Program, The Ohio State University, Columbus, OH, USA; 3 Integrated Biomedical Science Graduate Program, The Ohio State University, Columbus, OH, USA; 4 Department of Pediatrics, BSOM, East Carolina University, Greenville, NC, USA; 5 Experimental Therapeutics Program, The Ohio State University Comprehensive Cancer Center, College of Medicine, The Ohio State University, Columbus Ohio, USA

## Abstract

The Signal Transducer and Activator of Transcription (STAT) proteins comprise a family of latent transcription factors with diverse functions. STAT3 has well established roles in cell proliferation, growth and survival, and its persistent activation has been detected with high frequency in many human cancers. As constitutive activation of STAT3 appears to be vital for the continued survival of these cancerous cells, it has emerged as an attractive target for chemotherapeutics. We examined whether the inhibitory activities of bioactive compounds from cruciferous vegetables, such as Benzyl isothiocyanate (BITC) and sulforaphane, extended to STAT3 activation in PANC-1 human pancreatic cancer cells. BITC and sulforaphane were both capable of inhibiting cell viability and inducing apoptosis in PANC-1. Sulforaphane had minimal effect on the direct inhibition of STAT3 tyrosine phosphorylation, however, suggesting its inhibitory activities are most likely STAT3-independent. Conversely, BITC was shown to inhibit the tyrosine phosphorylation of STAT3, but not the phosphorylation of ERK1/2, MAPK and p70S6 kinase. These results suggest that STAT3 may be one of the targets of BITC-mediated inhibition of cell viability in PANC-1 cancer cells. In addition, we show that BITC can prevent the induction of STAT3 activation by Interleukin-6 in MDA-MB-453 breast cancer cells. Furthermore, combinations of BITC and sulforaphane inhibited cell viability and STAT3 phosphorylation more dramatically than either agent alone. These findings suggest that the combination of the dietary agents BITC and sulforaphane has potent inhibitory activity in pancreatic cancer cells and that they may have translational potential as chemopreventative or therapeutic agents.

## Background

Despite major advances in the detection and treatment of cancer in the past few decades, cancers of the pancreas are still rarely curable; the five-year survival rate of pancreatic cancer patients remains less than 5% [1]. Pancreatic cancers generally respond poorly to conventional treatment modalities such as chemotherapy and radiation therapy, necessitating the discovery and development of more effective means for their treatment [2].

The etiology of pancreatic cancer is poorly understood, but it involves the multi-stage development of aberrations in signaling pathways that affect cell growth and proliferation [3]. Recent studies have revealed the identities of several of these signaling proteins, including those associated with the ERK, AKT, mTOR and STAT3 pathways [4]. STAT3 is a member of the STAT family of transcription factors, which is transiently activated in response to cytokine and growth factor receptor stimulation [5-8]. Although it plays necessary roles in early development, the presence of STAT3 in the majority of adult tissue and cell types is mostly dispensable [9-11]. Constitutive STAT3 signaling has been implicated as a contributor to oncogenesis for its roles in stimulating cell proliferation, mediating immune evasion, promoting angiogenesis, and conferring resistance to apoptosis as induced by conventional therapies [12-16]. Persistent activation of STAT3 is frequently detected in a wide range of blood and solid tumors, including those of the pancreas [17-20]. Consequently, the inhibition of STAT3 by a variety of means has been demonstrated to exert a potent anti-cancer effect [15,21,22].

In the present study, we investigated the potential inhibitory effects of BITC and sulforaphane on persistent STAT3 activation in the PANC-1 pancreatic carcinoma cell line. BITC and sulforaphane are cruciferous vegetable-derived compounds which have been shown to inhibit chemically induced cancer in various animal models [23-28]. BITC is known to up-regulate the cyclin dependent kinase (Cdk) inhibitor p21^Waf1/Cip1^[29], induce a marked decline of Cdk1, cyclin B1 and cell division cycle 25B, and inhibit activation of nuclear factor κB (NF-κB) [30]. Sulforaphane has also been shown to inhibit NF-κB [31], histone deacetylase activity [32], and AKT phosphorylation [33], and it possesses several other anti-cancer activities [34]. It is presently unclear whether BITC or sulforaphane have any inhibitory effect on STAT3 activation in pancreatic cancers.

## Results

### Sulforaphane and BITC inhibit cell viability and induce apoptosis in PANC-1 pancreatic cancer cells

**Figure 1:**
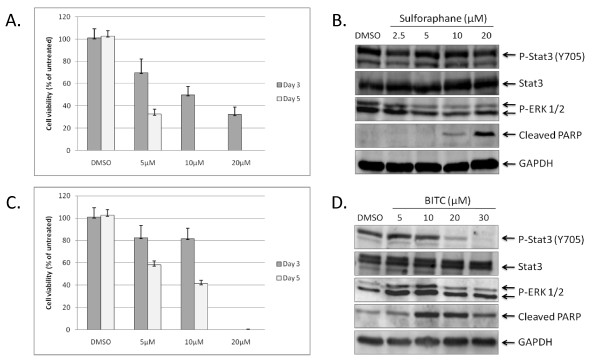
**The viability of PANC-1 pancreatic cancer cells is negatively impacted by sulforaphane and BITC**. (A) PANC-1 cells were treated with 5, 10, and 20 μM of sulforaphane for 3 and 5 days, after which MTT assays were used to assess cell viability. (B) Treatment of PANC-1 with 2.5-20 μM sulforaphane results in a marked increase in cleaved PARP, but otherwise has little to no impact on levels of phospho-ERK1/2 and pSTAT3. (C) Treatment of PANC-1 with 5, 10 and 20 μM BITC lowers PANC-1 viability to a lesser extent than that exhibited by sulforaphane. (D) BITC reduces levels of pSTAT3 in a dose-dependent fashion and increases levels of PARP cleavage, but has minimal effect on levels of phospho-ERK1/2.

We first examined the effect of sulforaphane and BITC treatment on the viability of PANC-1 as determined by MTT assays. At day 3 post-treatment, 5, 10, and 20 μM concentrations of sulforaphane inhibited approximately 30%, 50%, and 65% of cell viability respectively (Figure 1A). At day 5 post-treatment, 5 μM of sulforaphane was found to inhibit approximately 65% of cell viability; concentrations of 10 μM and 20 μM reduced viability below detectable levels (Figure 1A). We then investigated the effect of sulforaphane treatment on the phosphorylation status of STAT3. The activation of STAT3 is contingent upon phosphorylation of tyrosine residue 705 (Y705), an event typically preceded by the interaction of specific cytokines and growth factors with their cognate receptors [7,8]. Western blots were performed using lysates from PANC-1 cells treated with various concentrations of sulforaphane for 24 hours and a Y705-specific STAT3 antibody. Sulforaphane concentrations as high as 20 μM had no discernable effect on levels of phosphorylated STAT3 (pSTAT3) and minimal inhibitory activity on phosphorylated ERK 1/2, two closely related MAP kinases that are also regulated in part by tyrosine phosphorylation (Figure 1B) [35]. Concentrations of 10 and 20 μM sulforaphane were capable of inducing apoptosis in PANC-1 however, as evidenced by increased cleavage of poly-(ADP-ribose) polymerase (PARP), an early target of active caspases and a marker for apoptosis (Figure 1B) [36]. Taken together, these observations suggest that sulforaphane's inhibitory activities against PANC-1 are achieved by means independent of STAT3.

Similar experiments were performed with BITC concentrations ranging from 5 to 30 μM. The addition of BITC to the media likewise resulted in dose-dependent inhibition of the PANC-1 cells' viability, albeit to a comparatively lesser extent than that exhibited by sulforaphane (Figure 1C). In contrast to sulforaphane, Western blot analysis of cell lysates from BITC-treated samples revealed that levels of pSTAT3 dropped sharply at 20 μM BITC and were virtually undetectable at 30 μM (Figure 1D). Levels of phosphorylated ERK 1/2, however, remained relatively consistent. BITC was also found to induce apoptosis as determined by PARP cleavage (Figure 1D). These results suggest that BITC-mediated inhibition of PANC-1 may be at least partially dependent on suppression of activated STAT3.

### BITC treatment inhibits transcription of STAT3 target genes

**Figure 2:**
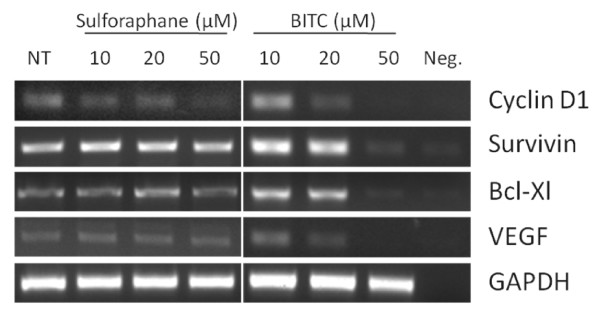
**BITC impacts expression of STAT3 target genes**. Non-quantitative RT-PCR shows BITC, but not sulforaphane, reduces transcription of several STAT3 target genes after 24 hours of treatment. NT = untreated. Neg. = no cDNA negative control.

To confirm our observations, we next performed non-quantitative reverse transcriptase PCR (RT-PCR) with cDNA generated from sulforaphane- and BITC-treated PANC-1 cells. We limited our investigation to well-characterized STAT3 target genes, such as the cell cycle regulator Cyclin D1, the anti-apoptotic proteins Survivin and Bcl-Xl, and the angiogenic mediator, vascular endothelial growth factor (VEGF) [37,38]. Sulforaphane treatments as high as 50 μM had marginal impact on the expression of these genes, whereas equimolar concentrations of BITC resulted in their suppression (Figure 2). These results again suggest that BITC-mediated inhibition of PANC-1 occurs through STAT3-dependent processes.

### BITC prevents phosphorylation of STAT3 by IL-6

**Figure 3:**
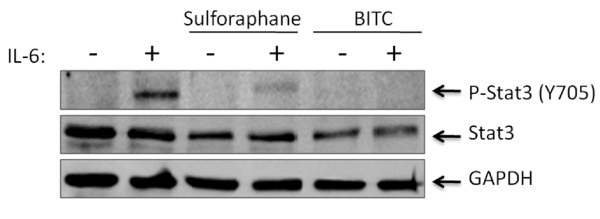
**Sulforpahane and BITC inhibit phosphorylation of STAT3 by IL-6**. The addition of 50 ng/ml of interleukin-6 stimulates STAT3 phosphorylation in MDA-MB-453 breast cancer cells. Pre-treatment of these cells with 50 μM sulforaphane or BITC resulted in the reduction or complete abrogation of pSTAT3 respectively.

Interleukin-6 (IL-6), a pleiotropic and proinflammatory cytokine, is known to be a potent activator of STAT3 [8]. Elevated serum levels of IL-6 are often associated with the pathogenesis of many cancers, including those of the pancreas and breast [39,40]. Following the observation that BITC could inhibit the phosphorylation of STAT3, we investigated if this compound was also capable of preventing STAT3 activation as mediated by IL-6. The already high endogenous levels of pSTAT3 in PANC-1 necessitated use of another cell line for these experiments. MDA-MB-453, a breast carcinoma cell line, was chosen due to its lack of detectable pSTAT3 in the absence of specific external factors. The addition of 50 ng/ml of IL-6 to the media of the MDA-MB-453 cells leads to rapid induction of pSTAT3, with maximum phosphorylation levels peaking within approximately 30 minutes (data not shown). When we pre-treated MDA-MB-453 cells with 50 μM concentrations of sulforaphane or BITC for two hours prior to the addition of 50 ng/ml IL-6 exposure, phosporylation of STAT3 was almost completely nullified by BITC (Figure 3). The molecular underpinnings for this inhibition remain to be elucidated, but given the short timeframe in which BITC is able to inhibit IL-6 mediated pSTAT3, BITC might be expected to somehow interfere with proper assembly or functioning of the IL-6 receptor complex, or perhaps disrupt the subsequent interaction of an IL-6-dependent kinase with STAT3. There is also the possibility that BITC directly interfaces with STAT3 itself. These hypotheses await further experimentation.

### The combination of sulforaphane and BITC is more potent than either agent alone

**Figure 4:**
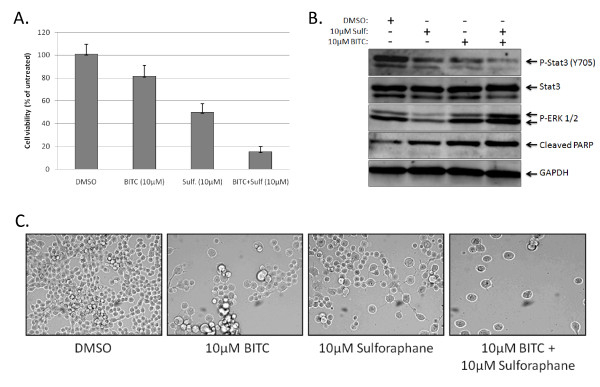
**The combination of sulforaphane and BITC inhibit cell viability to a greater extent than either agent acting separately**. (B) Western blot analysis of PANC-1 samples 24 hours post-treatment with the listed agents. (C) Sulforaphane and BITC alter the morphology and density of PANC-1 cells. Photographs were taken four days after treatment.

Finally, we investigated whether BITC and sulforaphane had synergistic qualities. We again performed MTT viability assays using 10 μM concentrations of sulforaphane and BITC, both alone and in conjunction. Following three days of treatment, 10 μM concentrations of BITC or sulforaphane reduced viability in PANC-1 cells by approximately 20% and 50% respectively (Figure 4A). PANC-1 cells treated with 10 μM BITC and 10 μM sulforaphane in conjunction displayed a greater than 80% reduction in viability (Figure 4A). This observation correlated with decreased levels of pSTAT3 and an increase in PARP cleavage as determined by Western blot (Figure 4B), as well as drastic alterations in morphology and overall cell number (Figure 4C).

## Discussion

Each year, approximately 40,000 individuals in the United States are diagnosed with pancreatic cancer [1]. Despite advancements in detection and treatment, the majority of these pancreatic cancer cases carry a grim prognosis. Even when detected early, cancer of the exocrine pancreas is rarely curable and has an overall survival rate of less than 4%. For patients with localized disease and small cancers (< 2 cm) with no lymph node metastases and no extension beyond the capsule of the pancreas, complete surgical resection can yield actuarial 5-year survival rates of only 18% to 24% [41]. For those patients with advanced cancers, the 5-year survival rate of all stages plummets to less than 1%, reflective of the poor response to chemotherapy and radiation therapy as conventionally used [2,42,43]. There is thus an urgent need to identify and develop more effective treatments for pancreatic cancer.

We identified a novel function of BITC to inhibit the tyrosine phosphorylation of STAT3 and prevent its induction by IL-6. BITC and sulforaphane were both capable of inhibiting cell viability and inducing apoptosis in PANC-1. Sulforaphane had minimal effect on the direct inhibition of STAT3 tyrosine phosphorylation, however, suggesting its inhibitory activities are most likely STAT3-independent. Conversely, BITC was shown to inhibit the tyrosine phosphorylation of STAT3, but not the phosphorylation of ERK1/2. These results suggest that STAT3 may be one of the targets of BITC-mediated inhibition of cell viability in PANC-1 cancer cells. Our results also suggest that the combination of bioactive compounds such as BITC and sulforaphane from cruciferous vegetables have potent inhibitory activity in human pancreatic cancer cells and may have potential as preventative or therapeutic agents.

## Materials and methods

### Cell Culture

PANC-1 pancreatic cancer cells and MDA-MB-453 breast cancer cells were acquired from ATCC. These cells were maintained in 1× Dulbecco's Modified Eagle's Medium (DMEM) supplemented with 10% fetal bovine serum (FBS) (Invitrogen), 4.5 g/L, L-glutamine, & sodium pyruvate (Mediatech) and 1% Penicillin/Streptomycin in cell culture incubators set at 37°C and 5% CO_2_.

### Western blot analysis

PANC-1 cells were treated with sulforaphane or BITC (Sigma-Aldrich) for 24 hours. In combination experiments, PANC-1 cells were treated with 10 μM of sulforaphane and/or 10 μM of BITC for 24 hours. For IL-6 experiments, MDA-MB-453 cells were pre-incubated with 50 μM sulforaphane or BITC for 2 hours before addition of 50 ng/ml IL-6 (Cell Sciences, Canton, MA). Western blots were conducted following SDS-PAGE of 100 μg of total lysate per sample. Membranes were blotted with antibodies specific for pSTAT3 Y705, STAT3, phospho-ERK1/2, cleaved PARP (Cell Signaling Tech) and GAPDH (Chemicon International Inc.). Membranes were analyzed with enhanced chemiluminescence Plus reagents and scanned with a Storm phosphorimager (Amersham Pharmacia Biotech Inc.).

### MTT Cell Viability Assay

PANC-1 cells were seeded in 96-well plates (6000 cells/well) in 10% FBS DMEM. After 24 hours, the media was replaced with 5% FBS DMEM and 0, 5, 10, and 20 μM sulforaphane or BITC for 3 or 5 days. In separate experiments, 10 μM of sulforaphane and BITC, either alone or in combination was added to PANC-1 cells for 3 days. At the end of each time point, 25 μl of MTT (Thiazolyl Blue Tetrazolium Bromide) was added to each well of the plate and incubated for 3.5 hours. Afterwards, 100 μl of N, N-dimethylformamide (Sigma-Aldrich) solubilization solution was added to each well. Plates were left at room temperature overnight to allow complete cell lysis, and read at 450 nm the following day. All experiments were repeated three times. Results are presented as averages with error bars representing one standard deviation.

### Reverse-transcriptase PCR

RNA was collected from PANC-1 cells with RNeasy Kits (Qiagen) following 24 hours of treatment with sulforaphane or BITC. cDNA was generated from 500 ng sample RNA using Omniscript RT (Qiagen). Two μl of cDNA was subsequently used for PCR. PCR amplifications were performed as follows: 5 min at 94°C followed by 25 cycles of [30 sec at 94°C, 30 sec at 55°C, 30 sec at 72°C] and a final extension at 72°C for 5 min. The PCR products were then run on 2% agarose gels, stained with ethidium bromide and visualized under UV light. Primer sequences and source information are available on request.

### Bright Field Microscopy

4 × 10^4 ^PANC-1 cells/well were seeded in six-well plates in 10% FBS DMEM and treated 24 hours later with sulforaphane and/or BITC. After four days of incubation, the cells were washed with PBS before being photographed under bright field microscopy at 100× magnification. Images were taken with a Model 9.0 Monochrome-6 camera on a computer equipped with Spot Advanced imaging software (Diagnostic Instruments Inc., Sterling Heights, MI). Three images of each treatment were taken from randomly chosen fields, and a representative image was selected for display in the figure.

## Competing interests

The authors declare that they have no competing interests.

## Authors' contributions

BH and WW carried out experiments for Western blotting and data analysis. SJ, SD, and BF carried out experiments for cell viability using MTT assays. LC, BH and JL contributed to the writing of the manuscript and participated in experimental designs. All authors read and approved the final manuscript.
